# Patient characteristics associated with the acceptability of teleconsultation: a retrospective study of osteoporotic patients post-COVID-19

**DOI:** 10.1186/s12913-023-09224-x

**Published:** 2023-03-08

**Authors:** Benedetta Pongiglione, Flaminia Carrone, Alessandra Angelucci, Gherardo Mazziotti, Amelia Compagni

**Affiliations:** 1 Centre for Research in Health and Social Care Management (CeRGAS), Bocconi University, Milan, Italy; 2 Department of Biomedical Sciences, Humanitas University, Pieve Emanuele, Milan, Italy; 3 Endocrinology, Diabetology and Medical Andrology Unit, Metabolic Bone Diseases and Osteoporosis Section, IRCCS Humanitas Research Hospital, Rozzano, Milan Italy; 4 Dipartimento Di Elettronica, Informazione e Bioingegneria, Politecnico Di Milano, Milan, Italy; 5 Department of Social and Political Sciences, Bocconi University, Milan, Italy

**Keywords:** Telemedicine, Acceptability, Osteoporosis, Chronic care, COVID-19

## Abstract

**Background:**

Due to the COVID-19 pandemic, teleconsultations (TCs) have become common practice for many chronic conditions, including osteoporosis. While satisfaction with TCs among patients increases in times of emergency, we have little knowledge of whether the acceptability of TCs persists once in-person visits return to being a feasible and safe option. In this study, we assess the acceptability of TCs across five dimensions for osteoporosis care among patients who started or continued with TCs after the COVID-19 pandemic had waned. We then explore the patient characteristics associated with these perceptions.

**Methods:**

Between January and April 2022, 80 osteoporotic patients treated at the Humanitas Hospital in Milan, Italy, were recruited to answer an online questionnaire about the acceptability of TCs for their care. The acceptability of TCs was measured using a modified version of the Service User Technology Acceptability Questionnaire (SUTAQ), which identifies five domains of acceptability: perceived benefits, satisfaction, substitution, privacy and discomfort, and care personnel concerns. Multivariable ordinary least squares (OLS) linear regression analysis was performed to assess which patient characteristics in terms of demographics, socio-economic conditions, digital skills, social support, clinical characteristics and pattern of TC use were correlated with the five domains of acceptability measured through the SUTAQ.

**Results:**

The degree of acceptability of TCs was overall good across the 80 respondents and the five domains. Some heterogeneity in perceptions emerged with respect to TCs substituting for in-person visits, negatively impacting continuity of care and reducing the length of consultations. For the most part, acceptability was not affected by patient characteristics with a few exceptions related to treatment time and familiarity with the TC service modality (i.e., length of osteoporosis treatment and number of TCs experienced by the patient).

**Conclusions:**

TCs appear to be an acceptable option for osteoporosis care in the aftermath of the COVID-19 pandemic. This study suggests that other characteristics besides age, digital skills and social support, which are traditionally relevant to TC acceptability, should be taken into account in order to better target this care delivery modality.

**Supplementary Information:**

The online version contains supplementary material available at 10.1186/s12913-023-09224-x.

## Background

The COVID-19 pandemic has caused profound disruptions globally to the delivery of healthcare services. Notably, it has affected the management of many chronic conditions as resources have been diverted to urgent care and as people have been less inclined to or prevented from attending healthcare facilities for fear of contagion [1, 2]. Since the early outbreak of COVID-19 in 2020, to guarantee continuity in the management of chronic conditions, many healthcare providers worldwide switched to remote consultations (teleconsultations [TCs]), in which the interaction between specialist or general practitioner and patients is mediated by some form of information technology-based platform [2, 3]. In Italy, one of the first and worst hit countries by the pandemic [4], telehealth services became forcibly widespread during the first outbreak, when the country adopted a strict lockdown policy. Previously, telehealth services had been rather uncommon and mostly treated with some suspicion by patients and healthcare professionals [5, 6].

In 2021, once the pandemic decreased in severity and people started to get vaccinated, the TC modality was maintained to limit the risk of crowding healthcare facilities [2] and is currently still in place in many countries for a select set of health conditions. Now that the pandemic is less pressing, it is of paramount importance to understand how patients continue to perceive the TC modality of delivering care, especially in comparison to in-person visits. There is still a limited understanding of which patient characteristics are associated with higher perceived benefits and the acceptability of TCs and therefore, which patients are more likely to continue with TCs even in times of non-emergency.

### Determinants of patients’ perceptions of telehealth and TCs

Perceptions of telehealth services before the COVID-19 pandemic were traditionally shaped by determinants such as age and digital literacy [6, 7]. For instance, a survey related to telehealth conducted in 15 European countries at the beginning of 2000 showed that interest in and acceptability of telehealth was significantly lower among older adults [6] and people with a lower education level [7]. In recent years, however, studies have shown much more heterogeneous results and an increase in satisfaction with telehealth, even among the elderly [8–10]. In a recent survey, European citizens ages 75 and over indicated their perception of telehealth as both making their lives easier and more difficult [9]. The ease of use of digital technology, convenience due to reduced travel time, increased access to healthcare professionals and even perception of improved health outcomes have all contributed to a high level of satisfaction with telehealth [8–10]. In these studies, though, some barriers were still highlighted, including patients’ inability to understand the technological side of telehealth, the need for social support when using new digital devices [9] and interactional challenges during video consultation (e.g., disruption to conversational flow and difficulty conducting examinations) [11].

With the COVID-19 pandemic, there has been an overall increase in patients’ degree of satisfaction with telehealth [12–15]. Nguyen et al. [14], for instance, found that during the COVID-19 outbreak, patients with diverse health conditions consistently reported 95–100% satisfaction rates with TCs in comparison to in-person visits. Similarly, studies in which TCs were targeted at patients with chronic cardiovascular [12] or rheumatologic [15] problems showed that during the early COVID-19 outbreak, TCs were greatly appreciated, and the majority of patients felt that without them, they would have stopped receiving pharmacological therapy or their health would have gotten worse. Most patients also indicated a willingness to continue with TCs in non-emergency situations [12].

### Telehealth in osteoporosis care

Osteoporosis is a common chronic condition worldwide that is linked to bone fragility; long-term pharmacological treatment is required for the prevention of further bone loss, deterioration of skeletal micro-architecture and disabling bone fractures [15, 16]. Given that osteoporotic patients have no real physical symptoms of the progression of the disease, one of the major issues in the treatment of osteoporosis is the low level of compliance with pharmacological therapy [16].

Consistent with studies of other chronic conditions, a review examining different models of telehealth for osteoporosis before the COVID-19 pandemic found limited evidence of the acceptability of these service modalities among osteoporotic patients and unclear evidence that telehealth services could improve drug therapy adherence [17]. More recently, higher acceptability of TCs among osteoporotic patients has been recorded. For instance, a study of 69 osteoporotic patients in Toronto found that participants were comfortable with TCs and perceived receiving a comparable quality of care to in-person visits [18]. They perceived the benefits of TCs in terms of convenience of timely care close to home, reduced burden of travel and costs and an enhanced sense of confidence in their osteoporosis specialist [18]. Patients also indicated the presence of some critical issues with TCs, including difficulties with sharing tests, conducting investigations through TCs and coordinating care with other healthcare professionals [18].

During the COVID-19 pandemic, osteoporotic patients experienced an increase in the use of TCs for their care [19] accompanied by an increase in the degree of acceptability of this service modality [17, 20]. A study based in the UK examined the perception of a virtual service for fracture risk assessment and fracture prevention advice during the COVID-19 pandemic [21]. The study showed that 90% of the 60 respondents rated their overall experience with the service as very good or excellent. Almost all of the respondents indicated that they would recommend the service to others and would continue the service after the end of the pandemic.

The previous findings mostly reported patients’ perceptions of TCs in times of emergency, while evidence of the acceptability of TCs after the emergency has waned is scant. This paper employs a modified version of the Service User Technology Acceptability Questionnaire (SUTAQ) [22–24] to assess how 80 Italian patients perceived the acceptability of TCs for their osteoporosis care post-COVID-19 across five different dimensions. We consider both patients that experienced their first TC during the early COVID-19 outbreak when in-person visits were unfeasible as well as patients who started with TCs later on when the pandemic was less pressing and in-person visits were again a feasible alternative. We then explore the patient characteristics correlated with such perceptions.

## Methodology

### Recruitment strategy, sample and data sources

This was a monocentric, retrospective study of patients at the Metabolic Bone Diseases and Osteoporosis Section of the IRCCS Humanitas Research Hospital in Rozzano, Milan, Italy. The inclusion criteria of the study were patients affected by osteoporosis who 1) were being treated with bone-active drugs, 2) had followed up by the time of recruitment (November 2021), and 3) had experienced at least one TC session. We retrospectively selected 102 subjects who met the inclusion criteria starting from those who had a follow-up visit in November 2021 and proceeding backward until June 2020. Patients were contacted via phone by the osteoporosis specialists of the Humanitas Hospital. Eighty accepted the invitation to participate in the study, three had died since their previous follow-up, 10 refused to participate and nine did not reply.

Of the 80 respondents, 38.7% had started TC visits between June 2020 and March 2021 (i.e., the emergency phase). This time period corresponded to the months immediately after the first COVID-19 outbreak (March–June 2020) and during the following two major pandemic outbreaks (i.e., September–December 2020 and January–March 2021) [25]. During this time, in-person visits were either not allowed or perceived as risky for fear of infection. The remaining 61.3% of the sample started with TC visits between April and November 2021 (i.e., the post-emergency phase), when in-person visits had become feasible again, the emergency had waned and patients (who were mostly vaccinated [25]) were less afraid of being infected while attending a healthcare facility. Of the 80 patients, 12.5% had explicitly asked to stop TC visits and return to in-person visits.

The TC service did not change during the study period. It consisted of a computer-based TC using a Google cloud platform (Humanitas Televisita Sicura Platform) developed by the Humanitas Hospital. Patients could connect with their osteoporosis specialists via video (through Google Meet). These specialists had been previously trained to use the TC platform by an internal board of technicians and experts in communication. The TC platform also allowed patients to share clinical data (e.g., the results of biochemical exams, previous clinical visits and imaging) with high safety standards for data protection. TCs were carried out interchangeably by two specialists (F.C. and G.M.), and osteoporotic patients received a TC every 6–7 months.

### Clinical data, questionnaire and analyses

For each recruited patient, demographics (e.g., age and gender), clinical characteristics (e.g., years since diagnosis of osteoporosis) and pattern of TC use (e.g., number of TCs before enrolment in the study) were retrieved from their clinical records. Additional patient characteristics were collected through a questionnaire. The questionnaire was administered to study participants via the Qualtrics online platform (Qualtrics, Provo, UT, USA; https://www.qualtrics.com). The informed consent form was first illustrated to the patients on the phone at the time of their recruitment by the Humanitas osteoporosis specialists and then sent via email (together with the link to the online questionnaire). Patients were instructed to carefully read the informed consent and to confirm they agreed with its contents by responding affirmatively to a statement contained in the first page of the online questionnaire. To guarantee the candidness of answers, the informed consent form explained to the patients that their answers would not be visible to their osteoporosis specialists.

The questionnaire included three parts. Part A asked questions about the socio-economic conditions of respondents, their digital skills and the presence of social support while using the TC platform. Table 1 reports descriptive statistics about these questions and the clinical characteristics of the sample of 80 patients. Additional file 1, instead, reports the overall baseline characteristics for the two groups (i.e., first TC in emergency phase and first TC in post-emergency phase) and shows that there was no significant difference between them.

**Table 1:** Descriptive statistics of patient sample and their characteristics

**Variable**	**% or mean (sd)**
***Demographics***	
Gender (female)	85.0
Age	
< 50	5.0
50–64	32.5
65–74	30.0
75 +	32.5
***Socio-economic conditions***	
Employment status	
Retired	61.3
Employed	32.5
Unemployed	6.3
Current job (or previous if retired or unemployed)^a^	
Business person, manager/academic, researcher, teacher	22.1
White collar/tradesman	42.9
Blue collar	16.9
Housewife	18.2
Education	
Low level	37.5
Middle level	30.0
High level	32.5
***Clinical characteristics***	
Charlson Comorbidity Index	7.3 (3.0)
No previous history of bone fracture	21.3
Bone fractures during observation period (i.e., TC)	7.5
Years since diagnosis of osteoporosis	10.8 (5.6)
Total length of pharmacological treatment for osteoporosis (years)^b^	8.2 (4.7)
Anti-osteoporosis pharmacological therapy	
Oral bisphosphonate	7.5
Denosumab	53.8
Teriparatide	32.5
Zoledronic acid	6.3
***Pattern of TC use***	
Date of first TC	
Emergency phase (June 2020-March2021)	38.7
Post-emergency phase (April 2021-November 2021)	61.3
Switch to TC from in-presence visits	51.3
N. TCs before enrolment in the study	2.2 (1)
In-person visits during TC	35.0
In-person visits during TC due to patient’s request	12.5
Use of TC for other chronic conditions	10.0
***Digital skills and social support***	
Social support in operating TC platform	55.0
IT skills	
Excellent/good	42.5
Mediocre	35.0
Very poor	22.5

*Legend*: *sd* standard deviation, *TC* teleconsultation

^a^ *N* = 77

^b^ *N* = 79

Part B of the questionnaire asked patients questions on the acceptability of the TC service for their osteoporosis care, while Part C collected qualitative suggestions for improvement of the TC service. For Part B, we adapted the Service User Technology Acceptability Questionnaire (SUTAQ, which has been previously validated in the literature [22–24] and translated into Italian [22]. Among the available questionnaires to evaluate telemedicine services, the SUTAQ is the third most used tool in the literature [26] and, unlike to the first two (i.e., Telehealth Usability Questionnaire, Telemedicine Satisfaction Questionnaire), is specifically designed to gather patients’ opinions about the acceptability of telemedicine and not about the usability of the technological platform associated with telemedicine or about patients’ overall satisfaction [26]. Thus, we considered the SUTAQ the most suitable instrument for our purposes. We obtained the translated and validated version of the SUTAQ [22] and adapted it to our purposes. Table 2 lists the modified SUTAQ questions and corresponding five domains of perceived benefits, satisfaction, substitution, privacy and discomfort, and care personnel concerns identified by Hirani et al. [23]. Answers used a 6-point Likert scale as previously done in the literature. We also added two new item questions (see NEW in Table 2) to the domains of perceived benefits and care personnel concerns by means of a qualitative interpretation of the domains.

**Table 2:** Modified SUTAQ questions and corresponding domain

**Question**	**Domain**
The teleconsultation service has made me more actively involved in my health	Perceived benefits
The teleconsultation service allows the specialists who are treating me to better monitor me and my osteoporosis	Perceived benefits
The use of the teleconsultation service can and should be recommended to people in a similar situation to mine	Perceived benefits
The teleconsultation service can certainly be a good addition to my regular health care	Perceived benefits
The use of the teleconsultation service has helped me to correctly follow the drug therapy prescribed for my osteoporosis. (NEW)	Perceived benefits
The teleconsultation service I received saved me time in that I did have to visit my osteoporosis specialist less often	Perceived benefits
The teleconsultation service I received increased my access health services for the treatment of osteoporosis	Perceived benefits
The teleconsultation service for osteoporosis treatment I received has helped me to improve my health status	Perceived benefits
Using the teleconsultation service has made it easier to get in touch with my specialist	Perceived benefits
The teleconsultation service has been explained to me sufficiently	Satisfaction
The teleconsultation service can be trusted to work appropriately	Satisfaction
Overall, I am satisfied with the teleconsultation service I received for the treatment of my osteoporosis	Satisfaction
The use of the teleconsultation service can be a replacement for the usual way of consulting in person	Substitution
Using the teleconsultation service is not as suitable as regular face to face consultation with the person treating me	Substitution
Using the teleconsultation service has allowed me to be less concerned about my health status	Substitution
The teleconsultation service I received interfered with my everyday routine	Privacy and discomfort
The teleconsultation service I received has invaded my privacy	Privacy and discomfort
Using the teleconsultation service made me feel uncomfortable, e.g. physically and/or psychologically	Privacy and discomfort
I am worried about the confidentiality of the private information being exchanged through the teleconsultation service	Privacy and discomfort
I am not convinced of the level of expertise of the specialists who monitor my health status through the teleconsultation service	Care personnel concerns
The teleconsultation service interferes with the continuity of care I am receiving (e.g., I do not see the same specialist each time)	Care personnel concerns
The use of the teleconsultation service has reduced the time that the osteoporosis specialist dedicates to me. (NEW)	Care personnel concerns

*Legend*: Added items are listed as (NEW)

To evaluate the fit of the original five-domain structure of the SUTAQ with our data, we applied confirmatory factor analysis (CFA). As shown in Additional File 2, factor loadings were above the 0.4 threshold commonly used in the literature with the exception of two items (invasion of privacy and interference with routine). This indicates that the five dimensions of acceptability proposed by the SUTAQ were largely found in our data as well. The CFA also confirmed our qualitative attribution of the two new items added to the SUTAQ domains of perceived benefits and care personnel concerns.

For each of the five domains, we calculated the mean values of the answers. The mean difference between domains was assessed using the t-test. We then applied multivariable ordinary least squares (OLS) linear regressions to assess the correlation between each domain of TC acceptability and patients’ characteristics.

Fifty-seven patients responded to the open question at the end of the questionnaire asking for aspects to improve the TC service. Of these, almost half (*n* = 26) of the respondents had no suggestion for improvement to report. For the remaining, we analysed the text of their answers inductively by identifying common themes across answers.

## Results

### Osteoporotic patients’ degree of acceptability of TCs

**Fig. 1:**
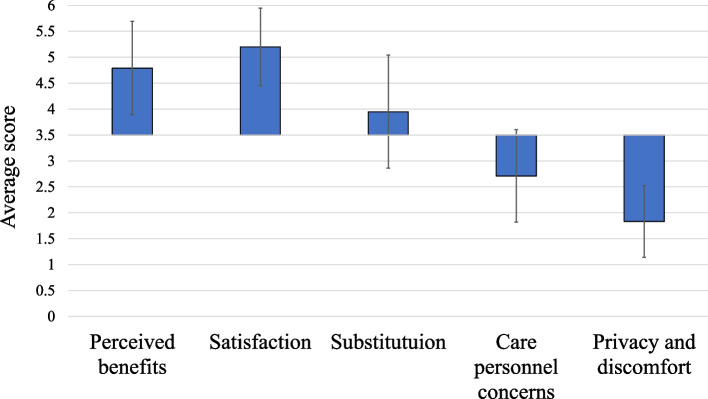
Average scores for the five domains of the modified SUTAQ. Legend: Average scores per domain are presented with respect to the overall average

Our analysis showed that patients overall accepted and appreciated the TC modality for their osteoporosis care, even after the COVID-19 emergency when in-person visits were a feasible alternative. Figure 1 illustrates the average scores of patients’ answers across the five domains of TC acceptability. For the domains of perceived benefits, satisfaction and substitution, a higher average value indicates higher acceptability, while for the domains of privacy and discomfort and care personnel concerns, a higher average value indicates lower acceptability. The first three domains displayed average scores between 4 and 5.5 (out of 6): 4.8 for perceived benefits, 5.2 for satisfaction and 4.9 for substitution. The other two domains had average scores between 1.5 and 3 (out of 6): 1.8 for privacy and discomfort and 2.7 for care personnel concerns. Being within these ranges indicated a good level of acceptability of TC among respondents.

**Fig. 2:**
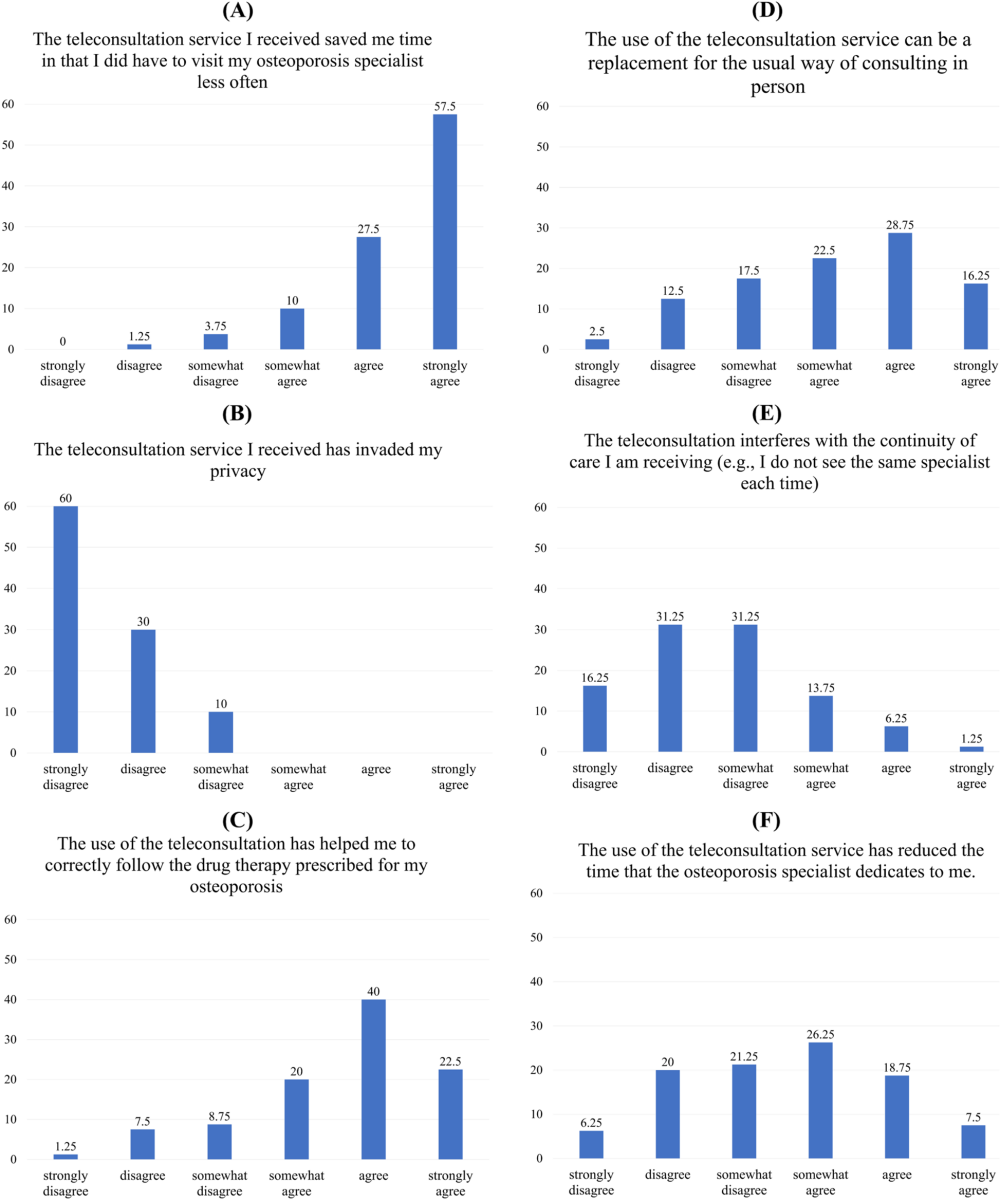
Exemplary skewed (left column) and distributed (right column) replies to items for each domain

Figure 2 reports the most and least skewed distributions of answers for select item questions, while the table in the Additional File 3 reports the mean, median and standard deviation for each questionnaire item. As evident from Fig. 2, some items displayed rather skewed distributions, indicating a homogeneously positive perception of TCs for those items. For instance, approximately 90% of respondents agreed or strongly agreed that TCs were time convenient (Fig. 2A) and did not invade their privacy (Fig. 2B). To a lesser extent, the question item we added to the SUTAQ exploring the impact of TCs on compliance with osteoporosis drug therapy showed an overall positive perception, with 62.5% of respondents agreeing or strongly agreeing that TCs have a positive impact on drug compliance (Fig. 2C).

Items that showed a higher variability across respondents were related to the perception of TCs as a replacement for in-person visits (Fig. 2D), that TCs could interfere with continuity of care (Fig. 2E) or that TCs reduce the time dedicated by the specialist to the patient (Fig. 2F). In this last case, for instance, over 50% of respondents at least somewhat agreed that TCs had reduced the length of the consultation with the specialist.

The heterogeneity of the perception of these items was also confirmed by the qualitative suggestions provided by respondents to the questionnaire’s open question. Respondents mainly commented on the poor substitutability of in-person visits with TCs, as demonstrated by the following quote:

In general, I am satisfied with TC. It makes you save time if it is about looking at exams or renewing drug therapies, but it must not completely substitute for in-person visits with the specialist for a matter of trust between doctor and patient. (Patient #31; emphasis added)

Other respondents indicated that not being able to see the same specialist during TC negatively impacted the continuity of their care and their overall perception of TCs. One patient commented:

In principle, I am fine with the idea of TC but, through the visits, there is the need to get to know better the specialist and create a trust relationship. If one [the patient] does not know who will be on the other side and maybe his competence… then it is not great. (Patient #19)

### Patient characteristics and acceptability of TCs for osteoporosis care

Table 3 reports the results of the OLS regression model expressing the correlation between each SUTAQ domain and patient characteristics. Table 4 synthesizes the most interesting results obtained through this analysis. Several characteristics that the literature previously found significant, such as age, the presence of social support with operating the TC platform and the level of digital skill, did not correlate with any dimension of the acceptability of TCs in this study. For context, 62.5% of our respondents were over 65 years old, 45% of them did not have any support to operate the TC platform and 57.5% of the sample perceived themselves as having mediocre or very poor digital skills.

**Table 3:** Multivariable OLS linear regressions for each SUTAQ domain

	**Perceived benefits**	**Satisfaction**	**Substitution**	**Care personnel concerns**	**Privacy and discomfort**
	ß (95% CI)	ß (95% CI)	ß (95% CI)	ß (95% CI)	ß (95% CI)
***Demographics***					
Gender					
Female	1.00	1.00	1.00	1.00	1.00
Male	0.18 (-0.56—0.91)	0.21 (-0.35—0.76)	0.6 (-0.23—1.43)	-0.41 (-1.09—0.26)	-0.50* (-1.03—0.04)
Age in years					
< 50	0.09 (-1.53—1.72)	-0.01 (-1.24—1.23)	-0.02 (-1.86—1.83)	0.1 (-1.39—1.60)	-0.46 (-1.65—0.73)
50–64	0.24 (-0.65—1.12)	0.03 (-0.64—0.70)	0.35 (-0.65—1.35)	-0.15 (-0.96—0.66)	-0.39 (-1.03—0.26)
65–74	1.00	1.00	1.00	1.00	1.00
75 +	-0.2 (-1.03—0.62)	-0.09 (-0.71—0.54)	-0.21 (-1.15—0.72)	-0.27 (-1.02—0.49)	0.01 (-0.60—0.61)
***Socio-economic conditions***					
Employment status					
Employed	1.00	1.00	1.00	1.00	1.00
Retired	0.22 (-0.64—1.09)	0.08 (-0.58—0.74)	0.17 (-0.81—1.15)	-0.15 (-0.94—0.65)	-0.54* (-1.17—0.10)
Unemployed	0.52 (-0.68—1.73)	0.47 (-0.45—1.38)	0.48 (-0.88—1.85)	-0.99* (-2.10—0.12)	-0.27 (-1.16—0.61)
Education					
Low	1.00	1.00	1.00	1.00	1.00
Intermediate	0.35 (-0.37—1.08)	0.26 (-0.29—0.81)	0.22 (-0.60—1.05)	-0.25 (-0.92—0.42)	-0.15 (-0.69—0.38)
High	0.44 (-0.34—1.22)	0.38 (-0.22—0.97)	0.17 (-0.71—1.05)	-0.29 (-1.00—0.43)	-0.15 (-0.72—0.42)
***Clinical characteristics***					
Charlson comorbidity index	0.03 (-0.08—0.14)	-0.02 (-0.11—0.06)	0.02 (-0.11—0.15)	0.01 (-0.10—0.11)	0.03 (-0.05—0.11)
History of bone fractures					
Never had	1.00	1.00	1.00	1.00	1.00
Had fractures	0.1 (-0.63—0.83)	0.41 (-0.15—0.96)	-0.02 (-0.84—0.81)	0.19 (-0.48—0.86)	-0.11 (-0.65—0.42)
Fractures during observation period					
No	1.00	1.00	1.00	1.00	1.00
Yes	0.11 (-0.61—0.82)	0.22 (-0.32—0.76)	0.51 (-0.30—1.32)	-0.45 (-1.11—0.20)	-0.18 (-0.71—0.34)
Years since osteoporosis diagnosis	0.04 (-0.01—0.09)	0.02 (-0.02—0.06)	0.04 (-0.01—0.10)	-0.02 (-0.06—0.03)	0 (-0.04—0.04)
Total length of pharmacological treatment for osteoporosis (years)	-0.05 (-0.12—0.01)	-0.04* (-0.10—0.01)	-0.09** (-0.17—-0.01)	0.06* (-0.00—0.12)	0.02 (-0.03—0.07)
Anti-osteoporosis treatment					
Teriparatide	1.00	1.00	1.00	1.00	1.00
Denosumab	-0.24 (-0.90—0.42)	-0.38 (-0.88—0.12)	-0.4 (-1.15—0.35)	0.37 (-0.24—0.97)	-0.01 (-0.50—0.47)
Oral bisphosphonate	-0.22 (-1.22—0.77)	0.07 (-0.69—0.83)	0.43 (-0.70—1.56)	0.11 (-0.81—1.02)	-0.16 (-0.89—0.58)
Zolendronic acid	-0.08 (-1.34—1.17)	0.18 (-0.78—1.13)	0.04 (-1.39—1.46)	0.16 (-0.99—1.32)	0.15 (-0.77—1.07)
***Pattern of TC use***					
Date of first TC					
June 2020-March 2021	1.00	1.00	1.00	1.00	1.00
April-November 2021	0.11 (-0.53—0.76)	0.41 (-0.08—0.90)	0.36 (-0.37—1.09)	-0.17 (-0.76—0.42)	-0.01 (-0.48—0.46)
Switch to TC from in-person visits					
No	1.00	1.00	1.00	1.00	1.00
Yes	-0.07 (-0.66—0.52)	-0.13 (-0.58—0.32)	0.06 (-0.60—0.73)	0.05 (-0.49—0.59)	-0.06 (-0.49—0.37)
N. TCs before enrolment in study	0.12 (-0.17—0.40)	0.22** (0.00—0.43)	0.08 (-0.24—0.40)	-0.06 (-0.32—0.20)	-0.12 (-0.33—0.08)
Face to face visits during TC					
No	1.00	1.00	1.00	1.00	1.00
Yes	-0.04 (-0.69—0.62)	-0.27 (-0.77—0.23)	-0.41 (-1.16—0.33)	0.14 (-0.46—0.75)	0.34 (-0.14—0.83)
Face to face visits during TC due patient’s request					
No	1.00	1.00	1.00	1.00	1.00
Yes	0.07 (-0.88—1.02)	0.48 (-0.25—1.21)	0.14 (-0.94—1.22)	-0.05 (-0.93—0.82)	-0.21 (-0.91—0.49)
Use of TC for other chronic conditions					
No	1.00	1.00	1.00	1.00	1.00
Yes	-0.37 (-1.25—0.50)	-0.53 (-1.20—0.13)	-0.19 (-1.18—0.81)	0.17 (-0.63—0.98)	0.27 (-0.38—0.91)
***Digital skills and social support***					
Social support in operating TC platform					
No	1.00	1.00	1.00	1.00	1.00
Yes	-0.02 (-0.73—0.69)	-0.22 (-0.76—0.32)	-0.27 (-1.08—0.53)	0.26 (-0.39—0.91)	-0.22 (-0.74—0.30)
Digital skills					
Excellent/good	1.00	1.00	1.00	1.00	1.00
Mediocre	0.19 (-0.54—0.93)	0.33 (-0.23—0.88)	0.17 (-0.66—1.00)	0.04 (-0.63—0.71)	0.3 (-0.23—0.84)
Very poor	0.39 (-0.85—1.63)	0.69 (-0.25—1.63)	0.49 (-0.91—1.90)	-0.4 (-1.54—0.74)	-0.01 (-0.92—0.90)

*Legend*: Significant results in bold; *** *p* < 0.01, ** *p* < 0.05, * *p* < 0.1

**Table 4:**
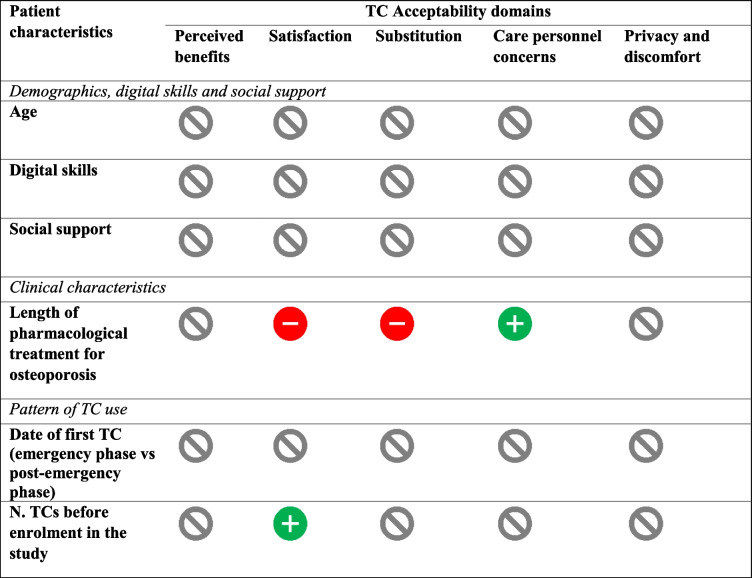
Selection of most significant correlations (or lack thereof) between patient characteristics and TC acceptability

*Legend*:  no significant correlation  significant negative correlation  significant positive correlation

We found some significant correlations elsewhere, although often the significance was weak. In particular, men were less concerned about issues of privacy and discomfort linked to TCs in comparison to women (ß = -0.50, *p* = 0.07). Considering that women are the most affected by osteoporosis, perhaps more caution should be used when dealing with osteoporotic women than men in TC. Retired patients were also less concerned in comparison to working individuals about the issues of privacy and discomfort (ß = -0.54, *p* = 0.095), and unemployed patients seemed less concerned with the quality of the relationship with the specialist than employed patients (ß = -0.99, *p *= 0.078).

The most interesting associations, though, referred to how suffering from a long-term condition such as osteoporosis affected the acceptability of TCs. Patients who had been treated for osteoporosis longer were the least satisfied with TCs (ß = -0.04, *p *= 0.086) and the least convinced of the substitutive capacity of TCs for in-person visits (ß = -0.09, *p* = 0.020). In addition, patients who had been treated for osteoporosis for longer were more concerned about the relationship with the specialist (ß = 0.06, *p* = 0.070). At the same time, patients who had experienced more TCs had greater satisfaction with TCs (ß = 0.22, *p* = 0.047), suggesting that the more patients become familiar with this service modality, the more acceptable it can become. We cannot completely exclude selection bias such that those satisfied with TC were more likely to continue with successive TCs. However, we controlled for patients requesting to stop TCs and go back to in-person visits (12.5% of study participants) and found no significant association between this dummy variable and the five acceptability domains.

Notably, the date of the first TC (emergency phase versus post-emergency phase) did not impact the acceptability of TCs across any of the five domains. This result was valid for all five acceptability domains and both the multivariable (Table 3) and univariable (i.e., mean difference; Additional File 4) analyses.

## Discussion

This study shows an overall good level of acceptability of TCs among patients with osteoporosis independently of whether they started TCs during the COVID-19 emergency phase or later on (when the emergency had waned and in-person visits returned as a feasible alternative). The findings indicate that for chronic conditions requiring regular follow-ups, TCs might be a valid care delivery modality even in non-emergency situations.

Despite this overall positive perception of TCs, some heterogeneity among patients was evident, especially with respect to the perception of the capacity of TCs to substitute completely for the kind of doctor–patient relationship that in-person visits engender. Specifically, several patients openly indicated concerns about TC negatively affecting the trust relationship with the specialist by reducing the continuity of care or consultation length.

Patient characteristics could only partially explain this heterogeneity in the acceptability of TCs. This might be due to unobservable individual characteristics, such as personality or attitudes, that we did not include in our model. For instance, Baudier et al. [27] showed that self-efficacy and personal innovativeness were relevant explanatory variables of patients’ intention to use telehealth services. Notably, in our study, old age, poor digital skills and a lack of social support with using the TC platform were not correlated with a lower acceptability of TCs. This confirms the trend observed in the literature of the increased acceptability of TCs for these kinds of patients even before the COVID-19 pandemic [8–10]. The pandemic may have just accelerated this process and, as such, further attenuated the relevance of these characteristics on the perception of TCs.

This study points to other characteristics that might be relevant to the acceptability of TCs, in particular, how long a person has received treatment for a chronic condition such as osteoporosis. Patients who have been treated for a longer amount of time may display more concern with TCs in terms of overall satisfaction, the possibility of TCs substituting for in-person visits and ensuring a high-quality relationship with the specialist. This alerts healthcare professionals to the fact that over time, people suffering from a chronic condition might feel fatigued and need a close relationship with their specialist in order to continue with the care of their chronic condition. In this, TCs might be perceived as less effective or satisfying in comparison to in-person visits.

The paper is a first attempt to measure the acceptability of TCs in the aftermath of the COVID-19 pandemic and is not exempt from limitations. The retrospective design and the small size of the study group could mean that the answers are not highly representative of osteoporotic patients. Moreover, some conditioning descending from patients being recruited to the study by their own osteoporosis specialists may have biased patients’ answers about the acceptability of TCs in a positive direction. We tried to attenuate this bias by explicitly informing patients that their specialist would not have access to their answers and including this information in the informed consent form that recruited patients had to sign.

## Conclusions

Our study provides useful insights into the acceptability of TCs for chronic conditions post-COVID-19. The significance of our findings lie in showing how TCs have become largely acceptable to categories of chronic patients who in the past were sceptical about this service modality, mainly for technological reasons. However, the study indicates that when these concerns are overcome, others might arise with respect to the quality of the doctor–patient relationship afforded by TCs. This suggests that as TCs and telemedicine in general become more widely adopted by healthcare systems, it is important to strengthen doctors’ and patients’ communication capacities in addition to their digital skills. In addition, it will be necessary to continue monitoring chronic patients’ perceptions of TCs in future years to understand how persistent this acceptability actually is and what affects it the most.

## Supplementary Information

**Additional file 1. **Descriptive statistics of respondents by date of first TC and their comparability.

**Additional file 2. **Confirmatory factor analysis showing loading factors for questions in the modified SUTAQ.

**Additional file 3. **Modified SUTAQ descriptors.

**Additional file 4. **Mean difference in SUTAQ acceptability domains between patients who experienced TC for the first time during and after the Covid-19 emergency phases.

## Data Availability

The dataset generated and analysed during the current study is not publicly available for privacy reasons, but it is available in an anonymised format from Bocconi University upon reasonable request of the corresponding author.

## Abbreviations

CFA: Confirmatory factor analysis
OLS: Ordinary least squares
SUTAQ: Service User Technology Acceptability Questionnaire
TC: Teleconsultation
