# Supplementary material for: Patient characteristics associated with the acceptability of teleconsultation: a retrospective study of osteoporotic patients post-COVID-19
Source: BMC Health Serv Res. 2023 Mar 8;23:230. doi: 10.1186/s12913-023-09224-x (PMC9994774; doi:10.1186/s12913-023-09224-x)
Supplement: Supplementary file 1 — Additional file 1. Descriptive statistics of respondents by date of first TC and their comparability. [file 12913_2023_9224_MOESM1_ESM.docx]

**Additional files of the article** ***Patient characteristics associated with the acceptability of teleconsultation: A retrospective study of osteoporotic patients post-Covid-19***

**Additional file 1:** Descriptive statistics of respondents by date of first TC and their comparability

|  | **First TC in emergency phase**  **(N=31)** | **First TC in post-emergency phase**  **(N=49)** | **p-value** |
| --- | --- | --- | --- |
| ***Demographics*** |  |  |  |
| Sex (female) | 87.1 | 83.7 | 0.676 |
| Age |  |  |  |
| <50 | 6.5 | 4.1 | 0.885 |
| 50-64 | 32.3 | 32.7 |  |
| 65-74 | 25.8 | 32.7 |  |
| 75+ | 35.5 | 30.6 |  |
| ***Socio-economic conditions*** |  |  |  |
| Employment status |  |  |  |
| Retired | 38.7 | 28.6 | 0.152 |
| Employed | 61.3 | 61.2 |  |
| Unemployed | 0.0 | 10.2 |  |
| Current job (or previous if retired or unemployed) § |  |  |  |
| Business person/manager/Academic position, researcher, teacher | 26.7 | 19.2 | 0.464 |
| White collar/tradesman | 43.3 | 42.6 |  |
| Blue collar | 20.0 | 14.9 |  |
| Housewife | 10.0 | 23.4 |  |
| Education |  |  |  |
| Low level | 35.5 | 38.8 | 0.362 |
| Middle level | 38.7 | 24.5 |  |
| High level | 25.8 | 36.7 |  |
| ***Clinical characteristics*** |  |  |  |
| Charlson Comorbidity Index | 7.0 (3.4) | 7.5 (2.7) | 0.466 |
| No previous history of bone fracture | 12.9 | 26.5 | 0.147 |
| Bone fractures during observation period (i.e., TC) (% yes) | 9.7 | 6.1 | 0.556 |
| Years since diagnosis of osteoporosis (s.d.) | 11.9 (5.1) | 10.0 (5.8) | 0.155 |
| Total length of pharmacological treatment for osteoporosis (in years) ǂ | 8.4 (4.7) | 8.1 (4.6) | 0.753 |
| Anti-osteoporosis pharmacological therapy |  |  |  |
| Oral bisphosphonate | 9.7 | 6.1 | 0.700 |
| Denosumab | 58.1 | 51.0 |  |
| Teriparatide | 29.0 | 34.7 |  |
| Zoledronato | 3.2 | 8.2 |  |
| ***Pattern of TC use*** |  |  |  |
| Switch to TC from in-presence visits | 35.5 | 61.2 | 0.025 |
| N TCs before enrolment in the study | 2.7 (1.0) | 1.8 (0.8) | <0.001 |
| In-person visits during TC | 41.9 | 30.6 | 0.301 |
| In-person visits during TC due to patient’s request | 16.1 | 10.2 | 0.435 |
| Use of TC for other chronic conditions (yes) | 6.5 | 12.2 | 0.400 |
| ***Digital skills and social support*** |  |  |  |
| Social support in operating TC platform | 51.6 | 57.1 | 0.628 |
| IT skills |  |  |  |
| Excellent/good | 32.3 | 49.0 | 0.334 |
| Average | 41.9 | 30.6 |  |
| Poor | 25.8 | 20.4 |  |

Legend: sd= standard deviation; TC=teleconsultation; §N=77; ǂ N=79
