# Supplementary material for: Patient characteristics associated with the acceptability of teleconsultation: a retrospective study of osteoporotic patients post-COVID-19
Source: BMC Health Serv Res. 2023 Mar 8;23:230. doi: 10.1186/s12913-023-09224-x (PMC9994774; doi:10.1186/s12913-023-09224-x)
Supplement: Supplementary file 2 — Additional file 2. Confirmatory factor analysis showing loading factors for questions in the modified SUTAQ. [file 12913_2023_9224_MOESM2_ESM.docx]

**Additional files of the article** ***Patient characteristics associated with the acceptability of teleconsultation: A retrospective study of osteoporotic patients post-Covid-19***

**Additional File 2**: Confirmatory factor analysis showing loading factors for questions in the modified SUTAQ

| **Domain** | **Items** | **Factor loadings** |
| --- | --- | --- |
| Perceived benefits | The teleconsultation service for osteoporosis treatment I received has helped me to improve my health status. | 0.845 |
|  | The teleconsultation service I received increased my access health services for the treatment of osteoporosis. | 0.822 |
|  | Using the teleconsultation service has made it easier to get in touch with my specialist. | 0.787 |
|  | The teleconsultation service I received saved me time in that I did have to visit my osteoporosis specialist less often. | 0.734 |
|  | The teleconsultation service allows the specialists who are treating me to better monitor me and my osteoporosis. | 0.760 |
|  | The teleconsultation service has made me more actively involved in my health. | 0.755 |
|  | The use of the teleconsultation service can and should be recommended to people in a similar situation to mine. | 0.730 |
|  | The use of the teleconsultation service has helped me to correctly follow the drug therapy prescribed for my osteoporosis. | 0.720 |
|  | The teleconsultation service can certainly be a good addition to my regular health care. | 0.706 |
| Satisfaction | Overall, I am satisfied with the teleconsultation service I received for the treatment of my osteoporosis. | 0.916 |
|  | The teleconsultation service can be trusted to work appropriately. | 0.827 |
|  | The teleconsultation service has been explained to me sufficiently. | 0.745 |
| Substitution | Using the teleconsultation service is not as suitable as regular face to face consultation with the specialist treating me. | -0.74 |
|  | The use of the teleconsultation service can be a replacement for the usual way of consulting in person. | 0.671 |
|  | Using the teleconsultation service has allowed me to be less concerned about my health status. | 0.639 |
| Care personnel concerns | I am not convinced of the level of expertise of the specialists who monitor my health status through the teleconsultation service. | 0.861 |
|  | The teleconsultation service interferes with the continuity of care I am receiving (e.g. I do not see the same specialist each time). | 0.443 |
|  | The use of the teleconsultation service has reduced the time that the osteoporosis specialist dedicates me. | 0.421 |
| Privacy and discomfort | Using the teleconsultation service made me feel uncomfortable, e.g. physically and/or psychologically. | 0.862 |
|  | I am worried about the confidentiality of the private information being exchanged through the teleconsultation service. | 0.455 |
|  | The teleconsultation service I received has invaded my privacy. | 0.392 |
|  | The teleconsultation service I received interfered with my everyday routine. | 0.241 |
