# Supplementary material for: Patient characteristics associated with the acceptability of teleconsultation: a retrospective study of osteoporotic patients post-COVID-19
Source: BMC Health Serv Res. 2023 Mar 8;23:230. doi: 10.1186/s12913-023-09224-x (PMC9994774; doi:10.1186/s12913-023-09224-x)
Supplement: Supplementary file 3 — Additional file 3. Modified SUTAQ descriptors. [file 12913_2023_9224_MOESM3_ESM.docx]

**Additional files of the article** ***Patient characteristics associated with the acceptability of teleconsultation: A retrospective study of osteoporotic patients post-Covid-19***

# **Additional file 3:** Modified SUTAQ descriptors

| **Items** | **Median** | **Mean** | **sd** | **min** | **max** |
| --- | --- | --- | --- | --- | --- |
| 1) The teleconsultation service I received saved me time in that I did have to visit my osteoporosis specialist less often. | 6 | 5.36 | 0.90 | 2 | 6 |
| 2) The teleconsultation service I received interfered with my everyday routine. | 2 | 2.20 | 1.32 | 1 | 6 |
| 3) The teleconsultation service I received increased my access health services for the treatment of osteoporosis. | 5 | 4.96 | 1.15 | 1 | 6 |
| 4) The teleconsultation service for osteoporosis treatment I received has helped me to improve my health status. | 5 | 4.55 | 1.23 | 1 | 6 |
| 5) The teleconsultation service I received has invaded my privacy. | 1 | 1.50 | 0.68 | 1 | 3 |
| 6) The teleconsultation service has been explained to me sufficiently. | 5 | 5.14 | 0.76 | 3 | 6 |
| 7) The teleconsultation service can be trusted to work appropriately. | 5 | 5.35 | 0.83 | 1 | 6 |
| 8) Using the teleconsultation service made me feel uncomfortable, e.g. physically and/or psychologically. | 2 | 1.78 | 1.06 | 1 | 6 |
| 9) I am not convinced of the level of expertise of the specialists who monitor my health status through the teleconsultation service. | 2 | 1.94 | 1.05 | 1 | 6 |
| 10) The teleconsultation service has made me more actively involved in my health. | 4 | 4.26 | 1.33 | 1 | 6 |
| 11) I am worried about the confidentiality of the private information being exchanged through the teleconsultation service. | 2 | 1.83 | 0.92 | 1 | 5 |
| 12) The teleconsultation service allows the specialists who are treating me to better monitor me and my osteoporosis. | 5 | 4.46 | 1.36 | 1 | 6 |
| 13) The use of the teleconsultation service can and should be recommended to people in a similar situation to mine. | 5 | 5.05 | 1.04 | 1 | 6 |
| 14) The use of the teleconsultation service can be a replacement for the usual way of consulting in person. | 4 | 4.11 | 1.36 | 1 | 6 |
| 15) The teleconsultation service can certainly be a good addition to my regular health care. | 5 | 5.20 | 0.82 | 2 | 6 |
| 16) Using the teleconsultation service is not as suitable as regular face to face consultation with the person treating me. | 3 | 3.05 | 1.36 | 1 | 6 |
| 17) Using the teleconsultation service has made it easier to get in touch with my specialist. | 5 | 4.66 | 1.14 | 1 | 6 |
| 18) The teleconsultation service interferes with the continuity of care I am receiving (e.g. I do not see the same specialist each time). | 3 | 2.66 | 1.17 | 1 | 6 |
| 19) Using the teleconsultation service has allowed me to be less concerned about my health status. | 4 | 3.80 | 1.36 | 1 | 6 |
| 20) The use of the teleconsultation service has helped me to correctly follow the drug therapy prescribed for my osteoporosis. (NEW) | 5 | 4.58 | 1.22 | 1 | 6 |
| 21) The use of the teleconsultation service has reduced the time that the osteoporosis specialist dedicates me. (NEW) | 4 | 3.54 | 1.37 | 1 | 6 |
| 22) Overall, I am satisfied with the teleconsultation service I received for the treatment of my osteoporosis. | 5 | 5.10 | 0.94 | 1 | 6 |
