# Supplementary material for: Patient characteristics associated with the acceptability of teleconsultation: a retrospective study of osteoporotic patients post-COVID-19
Source: BMC Health Serv Res. 2023 Mar 8;23:230. doi: 10.1186/s12913-023-09224-x (PMC9994774; doi:10.1186/s12913-023-09224-x)
Supplement: Supplementary file 4 — Additional file 4. Mean difference in SUTAQ acceptability domains between patients who experienced TC for the first time during and after the Covid-19 emergency phases. [file 12913_2023_9224_MOESM4_ESM.docx]

**Additional files of the article** ***Patient characteristics associated with the acceptability of teleconsultation: A retrospective study of osteoporotic patients post-Covid-19***

# **Additional file 4:** Mean difference in SUTAQ acceptability domains between patients who experienced TC for the first time during and after the Covid-19 emergency phases

|  | **Date of first TC** | | **Mean difference** | **p-value** |
| --- | --- | --- | --- | --- |
|  | Emergency phase  (June 2020-March 2021) | Post-emergency phase  (April 2021-November 2021) |  |  |
| Perceived benefits | 4.796 | 4.782 | 0.014 | 0.949 |
| Satisfaction | 5.129 | 5.238 | -0.109 | 0.529 |
| Substitution | 3.774 | 4.068 | -0.294 | 0.241 |
| Care personnel concerns | 2.828 | 2.639 | 0.189 | 0.357 |
| Privacy and discomfort | 1.815 | 1.832 | -0.017 | 0.915 |
